# In Vitro Antiviral Activity of Doxycycline against SARS-CoV-2

**DOI:** 10.3390/molecules25215064

**Published:** 2020-10-31

**Authors:** Mathieu Gendrot, Julien Andreani, Priscilla Jardot, Sébastien Hutter, Océane Delandre, Manon Boxberger, Joel Mosnier, Marion Le Bideau, Isabelle Duflot, Isabelle Fonta, Clara Rolland, Hervé Bogreau, Bernard La Scola, Bruno Pradines

**Affiliations:** 1 Unité Parasitologie et Entomologie, Département Microbiologie et Maladies Infectieuses, Institut de Recherche Biomédicale des Armées, 13005 Marseille, France; ma.gendrot@laposte.net (M.G.); o.delandre@gmail.com (O.D.); joelmosnier@orange.fr (J.M.); isabelle.fonta.09@gmail.com (I.F.); hervebogreau@yahoo.fr (H.B.); 2 Aix Marseille Univ, IRD, SSA, AP-HM, VITROME, 13005 Marseille, France; sebastien.hutter@univ-amu.fr; 3 IHU Méditerranée Infection, 13005 Marseille, France; miaguiabidou@gmail.com (J.A.); Priscilla.JARDOT@univ-amu.fr (P.J.); manon.boxberger@hotmail.fr (M.B.); Marion.LE-BIDEAU@ap-hm.fr (M.L.B.); isabelle.duflot@etu.univ-amu.fr (I.D.); rolland.clara@sfr.fr (C.R.); 4 Aix Marseille Univ, IRD, AP-HM, MEPHI, 13005 Marseille, France; 5 Centre National de Référence du Paludisme, 13005 Marseille, France

**Keywords:** COVID-19, SARS-CoV-2, doxycycline, treatment, prophylaxis, antiviral, anti-inflammatory

## Abstract

In December 2019, a new severe acute respiratory syndrome coronavirus (SARS-CoV-2), causing coronavirus disease 2019 (COVID-19), emerged in Wuhan, China. Despite containment measures, SARS-CoV-2 spread in Asia, Southern Europe, then in America and currently in Africa. Identifying effective antiviral drugs is urgently needed. An efficient approach to drug discovery is to evaluate whether existing approved drugs can be efficient against SARS-CoV-2. Doxycycline, which is a second-generation tetracycline with broad-spectrum antimicrobial, antimalarial and anti-inflammatory activities, showed in vitro activity on Vero E6 cells infected with a clinically isolated SARS-CoV-2 strain (IHUMI-3) with median effective concentration (EC_50_) of 4.5 ± 2.9 µM, compatible with oral uptake and intravenous administrations. Doxycycline interacted both on SARS-CoV-2 entry and in replication after virus entry. Besides its in vitro antiviral activity against SARS-CoV-2, doxycycline has anti-inflammatory effects by decreasing the expression of various pro-inflammatory cytokines and could prevent co-infections and superinfections due to broad-spectrum antimicrobial activity. Therefore, doxycycline could be a potential partner of COVID-19 therapies. However, these results must be taken with caution regarding the potential use in SARS-CoV-2-infected patients: it is difficult to translate in vitro study results to actual clinical treatment in patients. In vivo evaluation in animal experimental models is required to confirm the antiviral effects of doxycycline on SARS-CoV-2 and more trials of high-risk patients with moderate to severe COVID-19 infections must be initiated.

## 1. Introduction

In December 2019, a new severe acute respiratory syndrome coronavirus (SARS-CoV-2), causing coronavirus disease 2019 (COVID-19), emerged in Wuhan, China [1]. Despite containment measures, SARS-CoV-2 spread in Asia, Southern Europe, then in America and in Africa. As of 9 October 2020, according to the treatment guideline of the National Institutes of Health (NIH), there is no Food and Drug Administration-approved antiviral treatment against SARS-CoV-2 [2]. Several drugs are tested in the context of the DisCoVeRy trial [3] and a controversial early treatment that associates a combination of hydroxychloroquine and azithromycin has been used worldwide with a reduced risk of hospitalization, a reduced risk of death and a shorter duration of viral shedding [4,5,6,7,8,9,10]. Conversely, some studies showed that treatment of mild-to-moderate or mild-to-severe COVID-19 with hydroxychloroquine alone or in combination with azithromycin did not improve clinical status or duration of viral shedding in comparison with standard care [11,12,13,14,15]. Identifying effective, low cost antiviral drugs with limited side effects which are affordable immediately, especially for emerging countries, is urgently needed. An efficient approach to drug discovery is drug repurposing that consists of evaluating whether existing approved drugs can be efficient against SARS-CoV-2.

Doxycycline is a second-generation tetracycline with broad-spectrum antimicrobial [16] and anti-inflammatory activities [17]. Additionally, doxycycline was approved as prophylaxis against malaria by the Food and Drug Administration in 1994 and has been used since 2006 at the dose of 100 mg/day by the French military forces deployed in malaria-endemic areas [18].

Doxycycline also shows antiviral activity in vitro. This tetracycline derivative significantly inhibited the replication of the vesicular stomatis virus in vitro [19] and that of the dengue virus by inhibition of NS2B-NS3 serine protease [20,21,22]. Doxycycline showed inhibition of entry and replication of Chikungunya virus in Vero cell at 11 µM [23]. Using the in-silico method, doxycycline might be a potential inhibitor of Crimean-Congo hemorrhagic fever virus nucleoprotein, an essential protein in virus replication [24]. Additionally, doxycycline inhibited the early-stage replication of the porcine reproductive and respiratory syndrome virus, which causes respiratory disease, with EC_50_ (median effective concentration) of 0.25 µg/mL (about 0.5 µM) [25]. The current study evaluated the antiviral effect of doxycycline against SARS-CoV-2.

Doxycycline could be an attractive option for the treatment of COVID-19 [26]. Therefore, the activity of doxycycline was assessed in vitro against a clinically isolated SARS-CoV-2 strain and was compared with the activity of chloroquine.

## 2. Results

The cytotoxicity evaluation of doxycycline and chloroquine showed that the CC_50_ values were >100 µM for 48 h. The CC_50_ value of chloroquine is consistent with those previously described [27,28]. The antiviral effects of doxycycline against the clinically isolated SARS-CoV-2 strain (IHUMI-3) were concentration-dependent.

**Figure 1:**
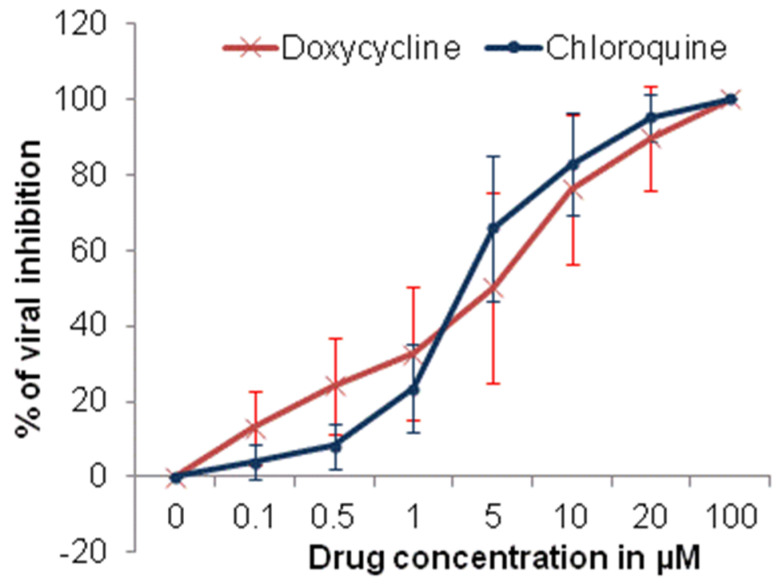
Comparative antiviral efficacy of doxycycline and chloroquine against SARS-CoV-2 infection in vitro (error bar represents standard deviation, 10 experiments).

**Figure 2:**
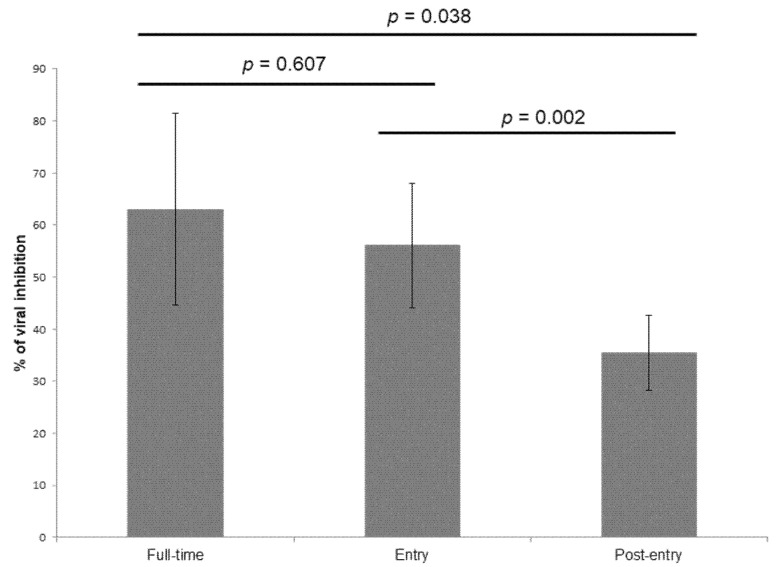
Antiviral activities of doxycycline at 5 µM against the SARS-CoV-2 IHUMI-003 strain in vitro. For full-time treatment, Vero E6 cells were pre-treated with doxycycline for 4 h and virus was then added for 48 h. For “entry” treatment, doxycycline was added to Vero E6 cells 4 h before viral infection and the virus-doxycycline mixture was replaced with fresh medium after 2 h post infection and was maintained for 46 h. For “post-entry” treatment, doxycycline was added 2 h post infection and was maintained for 46 h. Error bars represent the standard deviation of 10 experiments.

The median effective concentration (EC_50_) and 90% effective concentration (EC_90_) for doxycycline were 4.5 ± 2.9 µM and 23.5 ± 16.5 µM, respectively (Figure 1). The EC_50_ and EC_90_ for chloroquine were 3.2 ± 1.8 µM and 13.9 ± 6.4 µM, respectively (Figure 2). The EC_50_ value for chloroquine is consistent with previous results on Vero E6 cells at MOI of 0.2 to 0.25 [27,29].

The ratios C_max_/EC_50_ and C_max_/EC_90_ in blood for doxycycline were estimated at 0.75 and 0.07, respectively after an oral administration of doxycycline 100 mg, at 2.21 and 0.41 after an oral administration of doxycycline 200 mg and at 1.14 and 0.21 after intravenous administration of 100 mg of doxycycline.

Our results demonstrated that doxycycline interacted at both entry and post-entry stages of the SARS-CoV-2 infection in Vero E6 cells (Figure 2).

## 3. Discussion

The in vitro activity of doxycycline against the SARS-CoV-2 (EC_50_ = 4.5 µM) was consistent with those reported with hydroxychloroquine [27,29,30,31], with antimalarial drugs like chloroquine [27,28,29,32], amodiaquine [29,31,32], ferroquine [29], or mefloquine [29,31,33], antiviral agents like remdesivir [28,32,34] or lopinavir [32], and macrolides like azithromycin [34,35].

A daily oral uptake of 100 mg or 200 mg of doxycycline in healthy volunteers led to a C_max_ (maximum blood concentration) value of 1.7 and 5 µg/mL [36,37]. An intravenous doxycycline dose of 100 mg showed a C_max_ of 2.6 µg/mL [38]. The ratios C_max_/EC_50_ and C_max_/EC_90_ for doxycycline in plasma ranged from 0.75 to 2.21 and 0.07 to 0.41, respectively. The ratios C_max_/EC_50_ and C_max_/EC_90_ in plasma seem low to reach effective concentrations to inhibit SARS-CoV-2 in humans. However, in lungs, doxycycline were 2- to 4-fold higher than in plasma [39,40]. A daily oral uptake of 100 or 200 mg of doxycycline led to a C_max_ value from 3.4 to 20 µg/g of lungs. The ratios C_max_/EC_50_ and C_max_/EC_90_ for doxycycline in lungs ranged from 1.5 to 8.8 and 0.15 to 1.6, respectively.

Doxycycline interacted at both entry and post-entry stages of the SARS-CoV-2 infection in Vero E6 cells. Doxycycline inhibited in vitro both dengue virus replication by interaction with the dengue virus serine protease (DENV2 NS2B-NS3pro) and dengue viral entry by inhibition of the E2 envelope glycoprotein involved in virus entry [20,22]. Doxycycline interfered particularly with the adsorption and entry of the Chikungunya virus by interaction with the E2 envelope glycoprotein, but also inhibited viral replication in a lower manner [23]. However, doxycycline had no effect in vitro on the adsorption and entry of the vesicular stomatitis virus (VSV) and inhibited only viral replication [19]. Moreover, doxycycline enhanced the adsorption of the porcine reproductive and respiratory syndrome virus (PRRSV) and inhibited its replication after viral entry [25]. The inhibition of both entry and viral replication after SARS-CoV-2 entry is consistent with the results from combinatorial computational approaches. Docking analysis showed that doxycycline could strongly bind the spike protein (S) of SARS-CoV-2 [41]. The spike viral protein of SARS-CoV-2 used the ACE-2 receptor for entry [42]. Therefore, blocking these interactions offers a potential target for drug development. Moreover, doxycycline and more generally tetracyclines could bind the main protease (M^pro^) of SARS-CoV-2 [43,44]. This protein, also called 3C-like protease, is essential to conduct the replication cycle of SARS-CoV-2 by leading to the formation of non-structural proteins (NSPs) [45,46]. Moreover, Alexpandi et al. showed that doxycycline inhibited the RNA-directed 5′-3′ polymerase activity of SARS-CoV-2 RNA-dependent RNA polymerase (RdRp) (Alexpandi et al., personal data), a key enzyme for the replication of the SARS-CoV-2 [47], like remdesivir [48,49].

However, these results must be taken with caution regarding the potential use of antimalarial drugs in SARS-CoV-2-infected patients: it is difficult to translate in vitro study results into actual clinical treatment in patients. For instance, experts agree on the in vitro activity of chloroquine or hydroxychloroquine against SARS-CoV-2 but disagree on the hydroxychloroquine efficacy in COVID-19 treatments, which remains controversial [50,51]. In vivo evaluation in animal experimental models is required to confirm the antiviral effects of doxycycline on SARS-CoV-2.

Besides its antiviral activity, doxycycline has anti-inflammatory effects by decreasing the expression of various pro-inflammatory cytokines including interleukins 1 (IL-1), 6 (IL-6), and 8 (Il-8) and tumor necrosis factor-alpha (TNF-α) by macrophages [17,52] and chemokines including monocyte chemotactic protein 1, macrophage inflammatory protein 1α and 1β [53]. The immunomodulatory activity of doxycycline improved survival of septic mice with pulmonary inflammation [54]. Moreover, doxycycline is effective for the treatment of several chronic inflammatory airway diseases, including acute respiratory distress syndrome [55]. Doxycycline was the more effective tetracycline in the reduction of IL-6 and TNF-α in patients with dengue fever [56]. Doxycycline ameliorated pulmonary inflammation in a murine polymicrobial sepsis model by decreasing levels of IL-1β, IL-6 and TNFα in plasma and lungs [54]. Doxycycline improved the lung function and quality of life in chronic obstructive pulmonary disease (COPD) [57]. Levels of IL-6, IL-8 and TNFα were reduced in doxycycline-treated COPD patients. Moreover, doxycycline reduced neuroinflammation and preserved oxidative balance in traumatic brain injury-induced cognitive/motor impairments in rats due to its anti-inflammatory and free scavenging mechanisms [58]. Doxycycline could be a potential partner of COVID-19 therapies due to its anti-inflammatory effects.

Moreover, doxycycline is a bacteriostatic antibiotic that is active against the bacterial causes of community-acquired pneumonia, including *Streptococcus pneumoniae*, *Haemophilus influenzae*, and atypical pathogens such as *Mycoplasma pneumoniae*, *Chlamydia pneumoniae* and *Legionella spp* [59]. Doxycycline is used commonly for lower respiratory tract infection [60]. The use of doxycycline as antimalarial prophylaxis is associated with a reduced risk of Influenza-like illness among travelers [61]. Even without antiviral effects, doxycycline could be a potential partner of COVID-19 therapies due to anti-inflammatory effects and antibiotic effects that could prevent co-infections and superinfections.

Early treatment with doxycycline of 100 mg a day for 7 days (per os or intravenous) in 89 high-risk patients with moderate to severe COVID-19 infections was associated with early clinical recovery, decreased hospitalization and reduced mortality [62]. Another observational study on 54 high-risk patients in 3 long term care facilities in New York revealed that the use of a combination of 100 mg of doxycycline twice a day for 7 days and 400 mg of hydroxychloroquine twice a day on the first day and daily for the next 6 days was associated with a decrease in transfer to hospital and reduced mortality [63]. Results were compared and analyzed against the data observed in a long term care facility in Washington. However, these results must be confirmed by rigorous studies. Many clinical trials on doxycycline alone or in combination to treat COVID-19 are in progress [64,65,66].

## 4. Materials and Methods

### 4.1. Agent, Virus, and Cells

Stock solution of doxycycline hyclate (Sigma, Saint Louis, MO, USA) was prepared in methanol and diluted in Minimum Essential Media (MEM, Gibco, ThermoFischer, Waltham, MA, USA) in order to have 7 final concentrations ranging from 0.1 µM to 100 µM. Chloroquine diphosphate (Sigma) was used as comparator. The clinically isolated SARS-CoV-2 strain (IHUMI-3) [4] was maintained in production in Vero E6 cells (American type culture collection ATCC^®^ CRL-1586™) in MEM with 4% of fetal bovine serum and 1% glutamine (complete medium).

### 4.2. Cytotoxicity Assay

In vitro cell viability evaluation on the VERO E6 cell line was performed according to the method described by Mosmann with slight modifications [67]. Briefly, 10^5^ cells in 200 µL of complete medium were added to each well of 96-well plates and incubated at 37 °C in a humidified 5% CO_2_. After 24 h incubation, 25 µL of complete medium and 25 µL of each concentration of methylene blue, hydroxychloroquine or azithromycin were added and the plates were incubated for 48 h at 37 °C. After removal of the surpernatant, 100 µL of MTT (3-(4,5-dimethyl-2-thiazolyl) -2,5-diphenyl-2*H*-tetrazolium bromide, Sigma Aldrich, St Quentin Fallavier, France) solution (0.5 mg/mL in MEM without FBS) was then added to each well. Cells were incubated for 2 h at 37 °C. After incubation, the MTT solution was removed and 100 µL of dimethyl sulfoxide (DMSO) was added to dissolve the formazan crystals. Then, plates were shaken at 700 rpm for 10 min at 37 °C. The absorbance was measured at 570 nm using a TECAN Infinite F200 Microplate Reader. DMSO was used as blank. The 50% cytotoxicity concentration (CC_50_) was calculated with the inhibitory sigmoid E_max_ model, which estimated the CC_50_ through nonlinear regression by using a standard function of the R software (ICEstimator version 1.2) [68]. CC_50_ value resulted in the mean of 5 different experimentations.

### 4.3. Antiviral Activity Assay

Briefly, 96-well plates were prepared with 5.10^5^ cells/mL of Vero E6 (200 µL per well), as previously described. Doxycycline and chloroquine concentrations were added 4 h before infection. Vero E Cells were infected with IHUMI-3 strain at an MOI of 0.25. After 48 h post-infection, the replication was estimated by RT-PCR using the Superscrit III platinum one step with Rox kit (Invitrogene) after extraction with the BIoExtract SuperBall kit (Biosellal, Dardilly, France). The primers used were previously described [69]. The percentage of inhibition of SARS-CoV-2 replication was estimated for each drug concentration as following: (mean CT_drug concentration_ − mean CT_control 0%_)/(mean CT_control 100%_ − mean CT_control 0%_) × 100. EC_50_ (median effective concentration) and EC_90_ (90% effective concentration) were calculated with the inhibitory sigmoid E_max_ model, which estimated the EC_50_ and EC_90_ through nonlinear regression by using a standard function of the R software (ICEstimator version 1.2). EC_50_ and EC_90_ values resulted in the mean of 10 different experimentations.

### 4.4. Determination of the Inhibition Stage

Determining in vitro at what stage doxycycline is acting against the SARS-CoV-2 IHUMI-003 strain was assessed at a concentration of at 5 µM. For “full-time treatment”, Vero E6 cells were pre-treated with doxycycline for 4 h and virus was then added for 48 h. For “entry” treatment, doxycycline was added to Vero E6 cells 4 h before viral infection and the virus-doxycycline mixture was replaced with fresh medium after 2 h post infection and was maintained for 46 h. For “post-entry” treatment, doxycycline was added 2 h after post infection and was maintained for 46 h. The percentage of inhibition of SARS-CoV-2 replication by 5 µM of doxycycline was estimated for each drug concentration as following: (mean CT_drug concentration_ − mean CT_control 0%_)/(mean CT_control 100%_ − mean CT_control 0%_) × 100. The result was the mean of 10 different experiments.

### 4.5. Data Analysis and Interpretation

Results were estimated as a mean and standard deviation of 5 to 10 experiments. Selectivity index (SI) as a ratio of CC_50_/EC_50_ was estimated for doxycycline. The expected maximum blood concentration (C_max_) was estimated from the literature for doxycycline at doses commonly administered in oral or intravenous treatment. The ratios C_max_/EC_50_ and C_max_/EC_90_ were estimated to find out if the effective concentration in plasma to cure SARS-CoV-2 is achievable in humans. The ratios C_lung_/EC_50_ and C_lung_/EC_90_ were estimated from the data on doxycycline accumulation into the lung.

## 5. Conclusions

In conclusion, doxycycline showed a high in vitro antiviral effective activity against SARS-CoV-2 with IC_50_ (4.5 µM) compatible with oral uptake and intravenous administrations. Doxycycline interacted both on SARS-CoV-2 entry and in replication after virus entry. Besides its in vitro antiviral activity against SARS-CoV-2, doxycycline has anti-inflammatory effects by decreasing the expression of various pro-inflammatory cytokines and could prevent co-infections and superinfections due to broad-spectrum antimicrobial activity. Therefore, doxycycline could be a potential partner of COVID-19 therapies. However, these results must be taken with caution regarding their potential use in SARS-CoV-2-infected patients: it is difficult to translate in vitro study results into actual clinical treatment in patients. In vivo evaluation in animal experimental models is required to confirm the antiviral effects of doxycycline on SARS-CoV-2 and more trials of high-risk patients with moderate to severe COVID-19 infections must be initiated.
